# Attitudes of Swiss psychiatrists towards cannabis regulation and medical use in psychiatry: a cross-sectional study

**DOI:** 10.1186/s42238-023-00210-y

**Published:** 2023-12-06

**Authors:** Constantin Strube, Renato Davide Comazzi, Dimitri Löwinger, Reto Auer, Franz Moggi, Philippe Pfeifer

**Affiliations:** 1 University Hospital of Psychiatry and Psychotherapy, University of Bern, Bern, Switzerland; 2 Institute of Primary Health Care Bern (BIHAM), University of Bern, Bern, Switzerland; 3 Department of General Internal Medicine, University Hospital Bern, Inselspital, Bern, Switzerland; 4 Center for Primary Care and Public Health, Unisanté, Lausanne, Switzerland

**Keywords:** Cannabis, Medical use, Nonmedical use, Swiss psychiatrists, Regulation

## Abstract

**Background:**

Changes in regulation for cannabis for nonmedical use (CNMU) are underway worldwide. Switzerland amended the law in 2021 allowing pilot trials evaluating regulative models for cannabis production and distribution. Simultaneously, cannabis for medical use (CMU) in psychiatry is a growing issue, asked for by patients and being discussed by medical professionals.

**Methods:**

From December 2021 to February 2022, we conducted an online survey of psychiatrists in Switzerland. The survey comprised questions on attitudes towards regulative models for CNMU and towards prescribing CMU for mental disorders.

**Results:**

We contacted 2010 psychiatrists in Switzerland. A total of 274 (14%) participated in the survey. Sixty-four percent agreed to a regulated legalization of CNMU, and 89% would welcome pilot trials in Switzerland assessing models for regulating CNMU with those from a French-speaking region being more skeptical. Forty-nine percent of psychiatrists agree that CMU might have a therapeutic effect in mental disorders, but 50% agree that there is not enough scientific evidence yet. Participants working in an inpatient setting or in a French-speaking region as well as those with a longer duration of practice were more skeptical on CMU for mental health.

**Conclusions:**

Most surveyed Swiss psychiatrists favor the regulation of CNMU and the conduct of pilot trials. Despite little evidence and potential negative consequences, many participating Swiss psychiatrists agreed that cannabis could be efficacious in the treatment of some mental disorders advocating for more research in this topic.

**Supplementary Information:**

The online version contains supplementary material available at 10.1186/s42238-023-00210-y.

## Background

More than 10 years ago, Uruguay initiated the era of cannabis legalization and triggered the debate about changing the regulatory frameworks elsewhere. So far, the effects resulting from cannabis legalization are considered heterogeneous, and science acknowledges that it is too early to draw definitive conclusions on its impact on public and mental health (Hall et al. 2019). For example, after recreational legalization in Washington, no increase in cannabis use has been observed among adolescents and in young adults (Dilley et al. 2019). At the same time, cannabis-associated harms like depression or anxiety as well as accidental ingestion of cannabis products by children have increased in the North American adolescent population (Hamid et al. 2022). Further, the increasing prevalence of cannabis availability has reduced social perceptions of risks from cannabis use, although there is increasing knowledge on cannabis-associated risks for mental health (Bahji and Stephenson 2019; Chiu et al. 2022). Regarding cannabis for medical use (CMU), there is moderate evidence for a therapeutic application in chronic pain or multiple sclerosis (Stockings et al. 2018). Concurrently, there is still insufficient clarity for CMU prescription, possession of cannabis, education, and research-related issues for healthcare stakeholders (Perlman et al. 2021; Schlag et al. 2021). Also, a growing number of patients suffering from a mental condition report using cannabis as (self-) medication to alleviate mental health-related problems (Sarvet et al. 2018). However, there is insufficient evidence for providing recommendations on the therapeutic use of cannabinoids in mental disorders (Black et al. 2019).

In Switzerland, more than one-third of the population aged 15 years and older use cannabis at least once in their life (Health FOoP 2021). In 2013, the country of Switzerland lifted criminal sanctions for individuals found in possession of small amounts of the drug for their own use, but the consumption is still fined (Switzerland 2020). Previously, in 2008, only 36.7% of those who went to the polls supported the complete legalization of cannabis in a vote in Switzerland (Schweizer 2019). In March 2021, the Swiss Parliament changed the legislation to simplify access to cannabis for medical use. At the same time, the law of the Narcotics Act titled “experimentation article” was adopted, allowing scientific pilot trials for the dispensing of cannabis for nonmedical use (CNMU) in the next 10 years (Swiss Federal Chancellery 2021). Before this, the Federal Office of Public Health had initiated a representative survey among the general Swiss population, revealing that about two-thirds of respondents stated that they would vote yes or rather yes to a hypothetical referendum to legalize cannabis, whereas one-third would oppose legalization. The majority of participants voted for strong regulation regarding distribution, taxes, and a prohibition of advertising for cannabis products (Bosshard et al. 2021).

Overall, the situation in Switzerland concerning efforts to regulate the production, distribution, possession, and use of cannabis is dynamic and heterogeneous and follows the current trend of liberalization in other Western countries.

So far, there is not much knowledge on attitudes of physicians on cannabis regulation. One study found that general practitioners in Ireland rather support legalization of CMU but reject regulation for CNMU (Crowley et al. 2017). A recently published systematic review on physicians’ experiences, attitudes, and beliefs towards medical cannabis found that physicians specialized in addiction treatment seemed more skeptical compared to physicians in general (Ronne et al. 2021). Apart from general practitioners (GPs), psychiatrists are that professional group of physicians who is most abundantly confronted with cannabis-related (mental) health issues. One survey in Australian psychiatrists and trainees found that the participants were concerned about CMU to increase the risk for psychosis, apathy, and addiction. Further, the majority believed that CBD and THC could be prescribed for the treatment of pain and other nonpsychiatric conditions (Jacobs et al. 2019). In another survey, Colombian psychiatrists agreed that cannabis should be available for different medical conditions but expressed a rather low acceptance for the treatment of mental disorders with CMU (Orjuela-Rojas et al. 2021). As to date, a survey neither on attitudes towards regulation of CNMU nor on CMU for treating mental disorders have been conducted among psychiatrists in Switzerland or in Europe; we aimed at closing this gap with our study.

## Methods

### Survey design

We conducted a cross-sectional study with a descriptive mixed approach by using an online questionnaire to assess the self-reported knowledge, attitudes, and experiences of psychiatrists in clinical institutions and practices in Switzerland.

Our group designed and conducted this study’s questionnaire simultaneously and in cooperation with further surveys on attitudes towards CMU and CNMU among Swiss primary care physicians and pharmacists (Comazzi et al. 2022; Löwinger et al. 2022). A comprehensive and comparative analysis of those surveys is intended to be published soon. This present questionnaire comprised 31 items including qualitative descriptive questions, Likert-type questions, and multiple-choice questions. An informal group of a dozen GPs, psychiatrists, and psychologists completed the pilot questionnaire prior to the main study to obtain feedback in relation to the relevance and appropriateness of the items. After editing, the final version of the questionnaire was implemented in the web-based data management website and sent to the participants via email link.

First, the participants were questioned for demographic data. The items concerning their opinions on CMU and CNMU, their own perceived professional competence in this subject, and their interest in further education on the topic followed. Afterwards, we assessed the preferred ways of distribution of CNMU in case of legalization, and the last part concerned the participants’ attitudes, opinions, and management of prescribing CMU in psychiatry. Additionally, participants should answer items on two case reports concerning the medical and nonmedical applications of cannabis in a clinical situation (see Additional file 1). The structure of the questionnaire and the numbers of questions are shown in an additional table (see Additional file 2). As Switzerland is a multilingual country, the survey was conducted in German and French.

### Sample

Eligible participants were all practicing psychiatrists in Switzerland during the data collection period of ten weeks between December 2021 and February 2022. We excluded physicians or professionals that did not fulfill those criteria from the analysis. We included physicians with board certification for psychiatry in Switzerland. As Swiss psychiatric institutions also occupy physicians without board certification but with a long-standing experience in psychiatry, only participants with at least 2 years of experience in psychiatry were included in the analyses.

### Data collection

The first contact with all eligible participants was web-based via e-mail, containing a link to the study and information on the study objectives. Twelve psychiatric clinics in Switzerland, including four university hospitals (Bern, Basel, Genf, and Zürich) were contacted. Furthermore, cantons having a regional professional association of private psychiatrists (Baselland, Bern, Zürich, Waadt, Genf, Solothurn) were also contacted via email. The latter cantons represent 51.7% of the Swiss population in 2022. Each regional organization transferred the survey link to their actual members via email. Data collection was conducted by using the web-based data management website www.findmind.ch. The latter was used to build and administer the web-based questionnaires.

### Data analyses

We extracted the data from the data management website with transcript to an Excel (Microsoft^©^) file. Data analysis and extraction for presentation were then made with Excel. Survey results are reported as frequencies and percentages. It was possible for participants to finish the questionnaire without answering every question resulting in different numbers of given answers for each question. We did not exclude incomplete or unfinished questionnaires.

Further, we performed bivariate group comparisons and calculated a two-sample Wilcoxon rank-sum (Mann–Whitney) test for the following variables: age (< 50 years vs. > 50 years), sex, years of practice (< 10 years vs. > 10 years), setting (hospital vs. private practice), and language region (German vs. French). For the data analyses, we used Stata 17.0 (StataCorp. 2021, Stata Statistical Software: Release 17, College Station, TX, USA: StataCorp LLC). As null hypothesis, we assumed no differences between each tested pair of variables. To specify psychiatrist characteristics to predict attitudes towards research and regulation of cannabis, we conducted an ordered logistic regression analysis utilizing the “ologit” function of Stata and using all independent variables together at one time. Age and active professional years were used as ordinal variables. Setting was used as a categorical variable and the other demographic data as binary variables.

### Ethics statement

Ethical review and approval were waived for this study due anonymized surveys falling outside the Swiss Human Research Act.

## Results

Overall, 2010 Swiss psychiatrists were contacted, representing about 51% of the 3930 board-certified psychiatrists in Switzerland (Swiss Medical Association FMH 2021). Two-hundred and seventy-four (14%) of the contacted persons logged into the survey of which 194 (71%) did finish and 80 (29%) did not finish until the last item. Of those 80 partial finished responders, 7 participants answered more than 60%, and 21 answered more than 30% of total items. Further, of the 274 subjects who logged in, 17 (6.2%) did not answer any question, 160 (58.4%) did not answer every question, and 97 (35.4%) answered every question. The number of responses differs across the different questions due to the participants answering, and we report them for each question separately. Psychiatrists from 14 out of the 26 Swiss cantons participated in the survey. In total, 185 participants were Swiss board-certified psychiatrists. Non-board-certified psychiatrists (*n* = 66) were psychiatry-trained physicians working in Swiss psychiatric hospitals. Most of the participants were from the cantons of Bern (*n* = 61), Zürich (*n* = 41), Basel-Stadt and Baselland (*n* = 36), Genf (*n* = 20), or Waadt (*n* = 18). Additional participants were from Solothurn, St. Gallen, Aargau, Appenzell Ausserrhoden, Freiburg, Wallis, and Graubünden (*n* = 50 in total). Eighty-one percent were from a mainly German-speaking canton respectively. Table 1 shows the demographic data. For demographic information by language region and by responder status, see Additional file 3.

**Table 1:** Demographic characteristics of the participants who logged into the survey (*n* = 274)

	***n***	**%**
**Age**	***n* = 248**	
< 30 years	14	5.6%
30–40 years	50	20.2%
41–50 years	57	23.0%
51–60 years	67	27.0%
> 60 years	57	23.0%
Not specified	3	1.2%
**Sex**	***n* = 251**	
Women	102	40.6%
Men	142	56.6%
Other definition	3	1.2%
Not specified	4	1.6%
**Board-certified psychiatrist**	***n* = 251**	
Yes	185	73.7%
No	66	26.3%
**Active years in psychiatry**	***n* = 248**	
< 2 years	15	6.0%
2–5 years	21	8.5%
5–10 years	32	12.9%
11–15 years	39	15.7%
16–20 years	36	14.5%
> 20 years	103	41.5%
Not specified	2	0.8%
**Setting**	***n* = 248**	
Own practice	140	56.5%
Institution, mainly outpatient	55	22.2%
Institution, mainly inpatient	50	20.2%
Not specified	3	1.2%

**Fig. 1:**
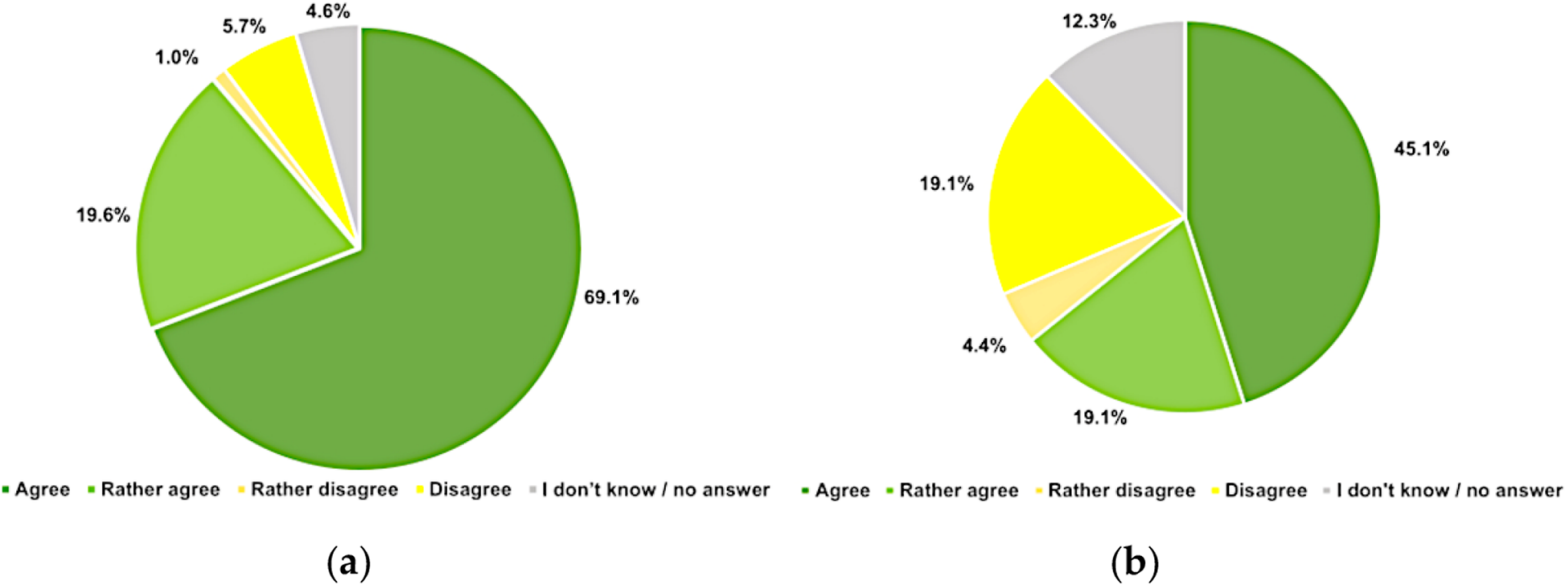
Attitudes of Swiss psychiatrists towards regulation and research. **a** Research on cannabis for nonmedical use. *n* = 194. **b** Regulated legalization. *n* = 204

Participating psychiatrists answered they agree on or rather agree on research efforts on CNMU by an 89% (172/194). In a hypothetical popular vote “Regulated legalization of cannabis use with effective health protection measurement,” they would agree or rather agree by a 64% (131/204), with 24% (48/204) disagreeing or rather disagreeing it (Fig. 1). Bivariate group comparisons yielded differences between German- and French-speaking participants for attitudes towards cannabis research (*p* = 0.0043) and regulation (*p* = 0.0021). Bivariate group comparisons across other major psychiatrist characteristics did not find significant differences between age groups, sex, duration of practice, practice setting, or board-certification status.

**Fig. 2:**
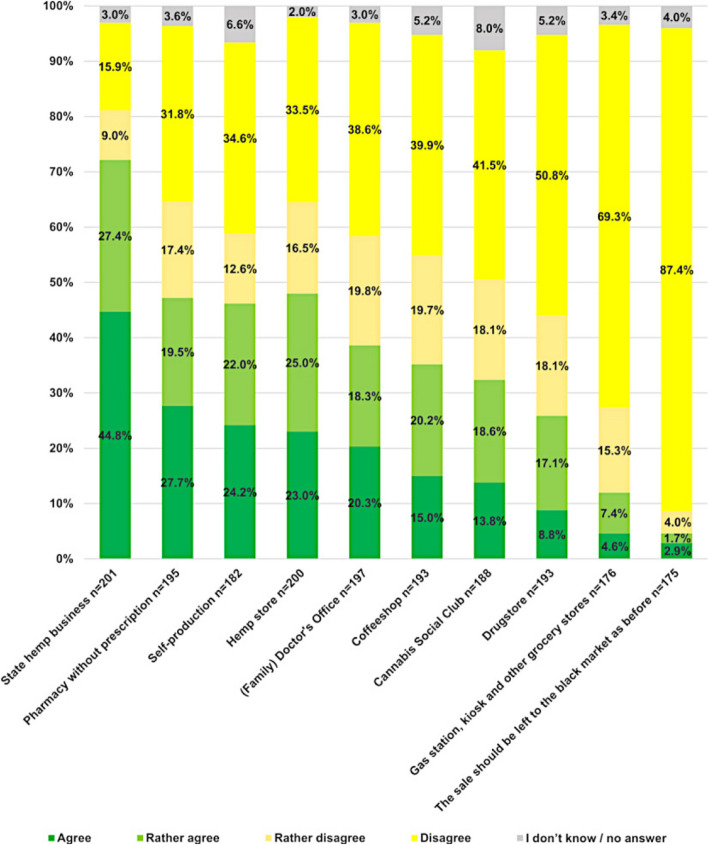
Psychiatrists’ attitudes towards suitable places of delivery for cannabis

Concerning the participants’ attitudes towards different options for purchasing cannabis in Switzerland, a majority of 72% (145/201) agreed with or rather agreed with cannabis distribution through state hemp businesses. Second, most agreed on or rather agreed on locations were pharmacies without prescription (47% (92/195)), self-production, and non-state hemp stores. Figure 2 shows further results to their attitudes.

**Fig. 3:**
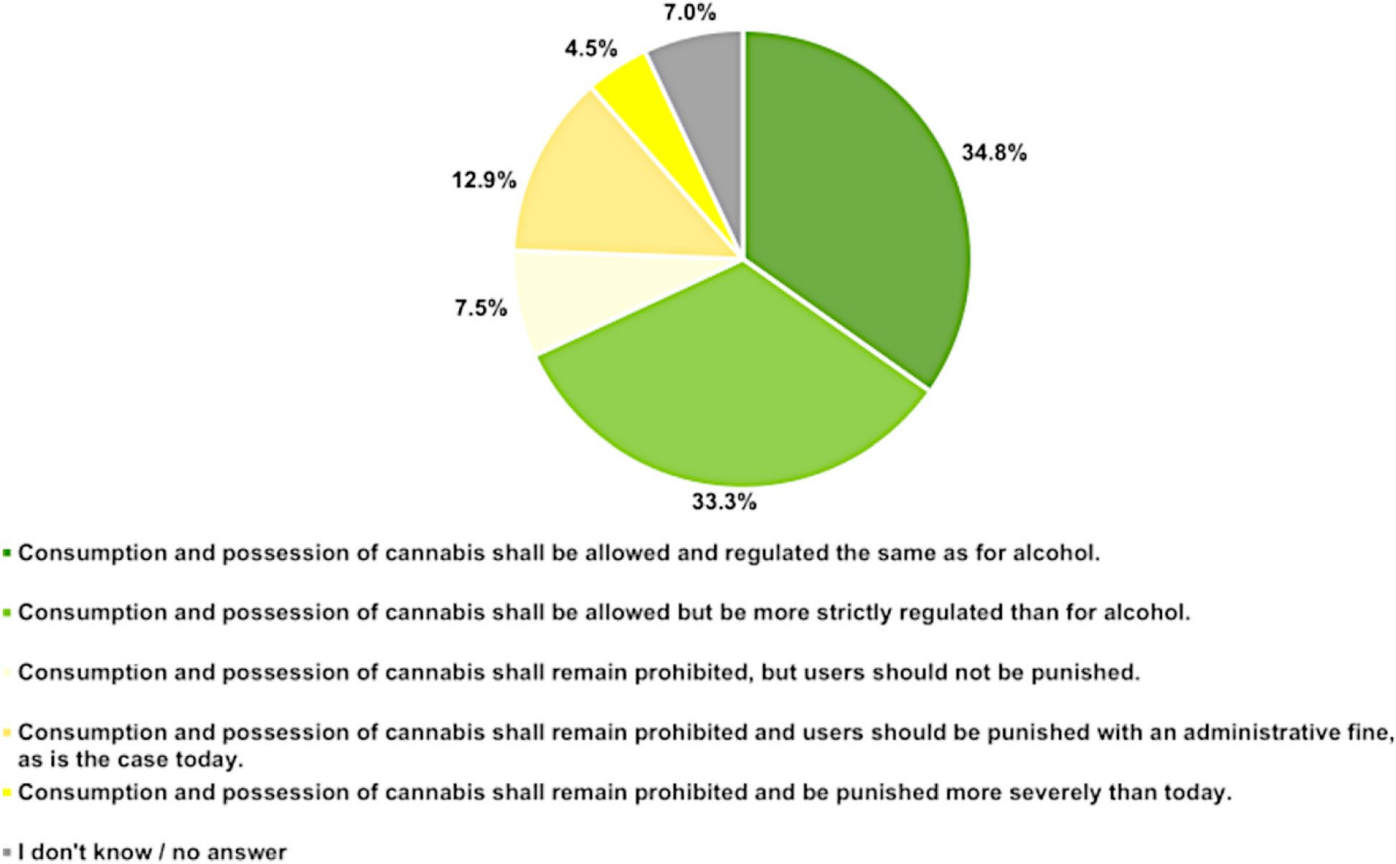
Attitudes towards possible regulations of possession and consumption of cannabis. *n* = 201

Sixty-eight percent (137/201) of the participants considered that cannabis should be regulated the same or more strictly than alcohol, as seen in Fig. 3. In contrast, 25% (64/201) of all participating psychiatrists voted that the possession of cannabis products should remain prohibited in Switzerland. The ordered logistic regression model yielded significant increase of agreement with an increasing number of active years (*p* = 0.031).

A vast majority agreed on or rather agreed on a regulated dispensing of cannabis at a 74% (150/203). On the other hand, the majority of participants disagreed on a regulated dispensing of heroin, cocaine, psilocybin, or MDMA (3,4-methylenedioxymethamphetamine) as can be seen in an additional figure (see Additional file 7).

**Fig. 4:**
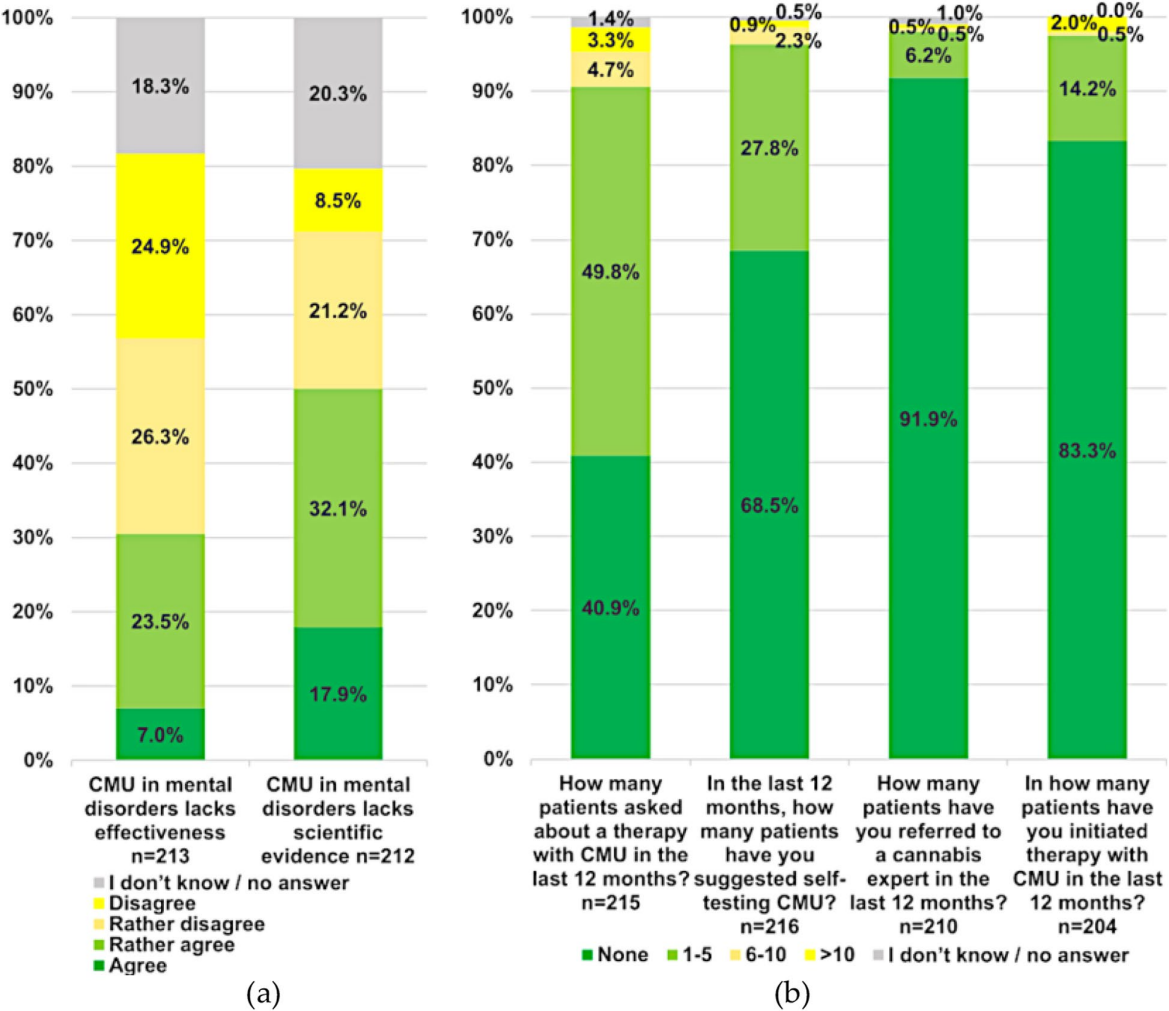
Attitudes and personal experience of CMU in psychiatry. **a** Attitudes of participants towards the application of CMU in psychiatry. **b** Professional experience with CMU in psychiatry

The surveyed psychiatrists also answered on their attitudes towards prescribing cannabis for mental disorders. Taken together, half of the participants stated that there was insufficient evidence for cannabis prescription in psychiatry (50%; 106/212). The ordered logistic regression model revealed significant disagreement for the number of active years (*p* = 0.019), the setting (*p* = 0.015), and the language region (*p* = 0.021). However, the majority of psychiatrists disagreed that cannabis would lack effectiveness in the treatment of mental disorders (51%; 109/212). Participants from the following subgroups were significantly more skeptical on positive effects of CMU on mental health: psychiatrists working in an institutional outpatient setting (*p* = 0.011) and those in the French-speaking regions (*p* < 0.001). The participating psychiatrists were also surveyed on their attitudes and experiences with CMU for psychiatric patients and rate the numbers of prescriptions. Figure 4 shows the results.

Participants further answered on how they rated the potential consequences of tetrahydrocannabinol (THC) and cannabidiol (CBD) on mental health. Most participants believed that THC had a negative effect on depression and psychosis, and that it could lead to dependency. At the same time, most participants had low or no concern about CBD prescription to have deteriorating effects on mental health (for details, see Additional files 4 and 5).

Furthermore, the participants were asked about their competence in cannabis prescription and counselling and in their interest on continuing education for cannabis. The minority of participants felt themselves competent to provide counselling on CNMU and CMU to patients. The majority was interested in continuing education on these issues. Details are shown in an additional figure (see Additional file 6). The questionnaire results on the two case reports concerning the medical and nonmedical applications of cannabis are shown in an additional file (see Additional file 1).

## Discussion

Overall, most participating psychiatrists support further research and demonstrate a rather liberal attitude towards a possible legalization of CNMU. Regarding CMU, they acknowledge the lack of evidence but not necessarily a possible lack of effectiveness, and they differentiate between THC and CBD in terms of possible negative side effects.

Former surveys on attitudes towards CMU and legalization were population based or focused on different medical specialists and other expert groups (Chiu et al. 2022; Crowley et al. 2017; Oldfield et al. 2020). We focused for the first time on psychiatrists as the core professional expert group who regularly advise patients with cannabis-related mental health problems. Compared to the total pool of Swiss psychiatrists, our sample corresponds to the demographic age and sex distribution as indicated in the official statistics of Swiss psychiatrists (Giacometti-Bickel et al. 2013). Our sample had a higher proportion of subjects from German-speaking cantons and of psychiatrists working in an institution than in a private practice (Swiss Medical Association FMH 2021).

Concerning CNMU, we found that most of the participating psychiatrists, and especially those who were German speaking, would vote in favor of a legalization, given that effective health protection measurements would be accounted for. Our findings are in line with a Swiss population survey from 2021 on cannabis regulation, which found that the German part of Switzerland was more in favor of pilot studies for CNMU than the French parts (Bosshard et al. 2021). The latter finding may correspond to the situation in the neighboring countries, where Germany has a more liberal course towards CNMU regulation compared to France. Further, a greater part of our sample supports research on this topic, which could help to stimulate the discussion with skeptic voices, who oppose mainly because of the expected increase in mental health problems and the fear of being a gateway drug for other substance use disorders (Luca et al. 2017). Likewise, the official position statements of professional associations such as the American Psychiatric Association tended to express concern about the potential negative consequences of cannabis use (Position statement on need to monitor and assess the public health and safety consequences of legalizing marijuana [press release] 2022).

A possible legalization of CNMU raises the issue of diverse options for selling and distribution. Most of the Swiss psychiatrists in our study were in favor of the model of a state hemp store for the distribution of nonmedical cannabis. This state hemp store model represents ownership by state corresponding to the regulated legalization model, as it is realized in the state of Québec in Canada. Simultaneously, the psychiatrists clearly rejected the profit-oriented ultraliberal distribution of cannabis provided in certain US states (e.g., Colorado) (Payan et al. 2021).

Concerning possible regulations, the psychiatrists in our study preferred moderate cannabis regulation measures to prohibition and no regulation. These findings are in line with recent Swiss survey data of both cannabis users and nonusers of the general population conducted in an urban environment in Switzerland (Znoj et al. 2021). In contrast, a previous survey in 2007 assessed the attitudes of 82 Swiss psychiatrists on cannabis risks in psychiatric patients and found controversial opinions with rather categorical prohibitive or permissive positions (Zullino et al. 2008). The authors concluded that attitudes in psychiatrists resembled to those found in the population, indicative more of an ideological than empirical approach to the cannabis issue. We found the amount of active years as a variable that is associated with attitude towards cannabis regulation with older psychiatrists tending to agree for a more liberal approach. Interestingly, this finding contrasts with attitudes in the general population where younger people were more open to cannabis regulation and may reflect the professional and educational experience of the former with cannabis (Bosshard et al. 2021).

Likewise, the psychiatrists in our study showed a more liberal attitude compared to the survey data of Irish GPs, where the majority did not support a drug policy of cannabis decriminalization (Crowley et al. 2017). Also, the participating psychiatrists in our survey did not share the skepticism of surveyed addiction treatment specialists towards a regulative approach to distribute cannabis for CNMU (Ronne et al. 2021).

Members of our study group have also conducted a survey similar to the present in Swiss primary care physicians. Comparing both groups, psychiatrists seemed more liberal than the primary care physicians as the trend towards a legalization of CNMU was higher with 68% of psychiatrists compared to 56% of primary care physicians (Comazzi et al. 2022).

Regarding the use of cannabis for mental disorders, the rating of most of our psychiatrists corresponds to the scientific literature according to which evidence is too scarce to legitimate prescribing of cannabis for mental disorder (Black et al. 2019). Here, we found that those with more years of psychiatric experience, those working in an institution and those from a French canton are more skeptical than their counterparts. In contrast, psychiatrists answered that their reservation towards cannabis prescriptions for psychiatric conditions was not mainly due to an estimated lack of effect. Regarding this, our survey showed a significant difference between the respondents from the German-speaking part in contrast to those from the French-speaking parts with the latter being more skeptical about the effectiveness. This matches with our findings about attitudes towards legalization mentioned above. Interestingly, the participants in our study who work in an institution are more skeptical than those working in their own practice.

In terms of possible negative consequences of the consumption, participants rated that CBD might have a less negative impact on mental health than THC. The latter was rated to particularly worsen psychosis and memory, which is in line with the previous surveys with psychiatrists (Jacobs et al. 2019; Gage et al. 2016). There is one former survey, which asked Colombian psychiatrists about their knowledge and attitudes about cannabis in medicine. The latter agreed that insomnia and anxiety are mental health conditions, for which cannabis use might improve symptoms (Orjuela-Rojas et al. 2021).

About one-third of the psychiatrists felt rather insecure about providing counselling about CMU and had a strong interest in continuing education on this issue. These findings are in line with the attitudes of Australian psychiatrists being unsure of indications for cannabinoid prescription. The authors concluded that further education about CMU seem to be necessary (Jacobs et al. 2019). More than half of the psychiatrists stated that they had already been asked by patients to initiate treatment with CMU. Additionally, a small group of participants recommended cannabis self-medication or prescribed cannabis for mental health to their patients. This attitude corresponds to an increasing interest and belief of patients and the public in the use of cannabis to treat mental health-related problems (Gilman et al. 2022).

Our study has several limitations. Firstly, the surveyed psychiatrists were not a representative sample of all Swiss psychiatrists. Referring to the statistics of the Swiss Medical Association (Foederatio Medicorum Helveticorum; FMH) in 2021, there have been 3930 board-certified psychiatrists in Switzerland (Swiss Medical Association FMH 2021). Therefore, the 185 respondents from most Swiss cantons represent about 4.7% of all Swiss psychiatrists.

Secondly, since we only achieved a response rate of 13.8%, the sample may be considered a selected group of psychiatrists potentially biased, both towards a more negative or a more positive attitude, regarding cannabis legalization and medical use in psychiatry. Also, we did not conduct a sample size estimation so that the power of our statistical analyses is unknown. Our survey sample is representative for age and sex but does not completely match with the Swiss pool of all psychiatrists for language region or setting, and therefore, findings related to these two variables might be not representative for the population of Swiss psychiatrists. Especially, we have not considered the Italian- and Romansh-speaking psychiatrists as representatives of the two other Swiss national languages. Further, as it was possible for the participants to drop out of the survey before finishing and it was possible to skip questions, there might be a loss of information in either direction. At last, these findings cannot be generalized to other countries in Europe, America, or elsewhere as access to cannabis, practice of prescription, and legal frameworks substantially differ between countries.

## Conclusions

We found that most surveyed Swiss psychiatrists favor the conduct of pilot trials testing regulated nonmedical cannabis use. Despite few evidence and potential negative consequences of medical cannabis use for the treatment of mental health problems, participating Swiss psychiatrists advocate for more research in this field. Taken together, our study was the first to survey psychiatrists on regulative aspects of cannabis use and for CMU in mental health. It yielded that Swiss psychiatrists tend to plead for a legal approach to regulate CNMU, and that they are aware of missing evidence to use cannabis for the treatment of psychiatric conditions.

### Supplementary Information

**Additional file 1. **Case reports and associated questions; A file containing the texts, questions and results concerning both case reports, translated into English.

**Additional file 2. **Structure of the questionnaire; A table showing the chapters of the survey and the associated questions’ numbers.

**Additional file 3. **Demographics of subgroups; A table showing the demographics by canton and the demographics by responder status.

**Additional file 4. **Swiss psychiatrists’ opinion on emergence of consequential diseases with THC use; A stacked bar chart showing the participants’ opinion of possible consequential diseases associated with consumption of THC.

**Additional file 5. **Swiss psychiatrists’ opinion on emergence of consequential diseases with CBD use; A stacked bar chart showing the participants’ opinion of possible consequential diseases associated with consumption of CBD.

**Additional file 6. **Competency and interest in continuing education of Swiss psychiatrists concerning CNMU and CMU; A stacked bar chart showing the personally perceived competency and interest of continuing education concerning CNMU and CMU.

**Additional file 7. **Support of regulated dispensing of various drugs; A stacked bar chart showing the participants’ support of regulated dispensing of various drugs.

**Additional file 8. **Bivariate analyses; A table with the quantitative results of the bivariate analyses.

**Additional file 9. **Ordered logistic regression analyses; A table with the quantitative results of the ordered logistic regression analyses.

## Data Availability

The datasets used and analyzed during the current study are available from the corresponding author on reasonable request. Data which could be used as secondary identifiers will be removed beforehand. The data are not publicly available due to privacy concerns.

## Abbreviations

CNMU: Cannabis for nonmedical use
CMU: Cannabis for medical use
GP: General practitioner
THC: Tetrahydrocannabinol
CBD: Cannabidiol
MDMA: 3,4-Methylenedioxymethamphetamine
FMH: Foederatio Medicorum Helveticorum
