# Supplementary material for: Attitudes of Swiss psychiatrists towards cannabis regulation and medical use in psychiatry: a cross-sectional study
Source: J Cannabis Res. 2023 Dec 6;5:40. doi: 10.1186/s42238-023-00210-y (PMC10699035; doi:10.1186/s42238-023-00210-y)
Supplement: Supplementary file 1 — Additional file 1. Case reports and associated questions; A file containing the texts, questions and results concerning both case reports, translated into English. [file 42238_2023_210_MOESM1_ESM.pdf]

Case vignette A: Mrs. A. 45 y. / “Cannabis, CBD and THC for therapy in mental disorders

"Ms. A. is a 45-year-old office worker. She has been in treatment with you for depression and anxiety for several years. Previous attempts at therapy with antidepressants have brought only slight relief. Furthermore, the patient prefers herbal substances and does not want to take any more antidepressants. Mrs. A. rarely drinks alcohol and is a non-smoker. She smokes one joint with cannabis 2-3 times a week, which has a clear mood-lifting and anxiety-relieving effect.

Ms. A. does not want to continue smoking cannabis and is not comfortable with having to obtain cannabis on the illegal black market. She therefore wishes to try therapy with medical cannabis/cannabinoids.

As a specialist in psychiatry, how do you proceed with this request for a therapy trial with medical cannabis/cannabinoids? How do you assess the situation?

Personal attitude regarding a therapy attempt in Mrs. A.

	**Ø**	**agree 1**	**rather agree 2**	**rather disagree 3**	**disagree 4**	**do not know / no answer 5**
Personally, I am positively disposed towards a therapy trial of Mrs. A. with medical cannabis/cannabinoids	Ø: 2.49 Σ: 228	62 27.19%	64 28.07%	44 19.3%	45 19.74%	13 5.7%

Possible procedures in relation to case vignette A

	**Ø**	**agree 1**	**rather agree 2**	**rather disagree 3**	**disagree 4**	**do not know / no answer 5**
I advise against trying therapy.	Ø: 2.95 Σ: 205	38 18.54%	40 19.51%	33 16.1%	82 40%	12 5.85%
I recommend that the patient try CBD therapy on her own (privately) with less than 1% THC; re-consultation if there is subsequently a continued desire for medical prescription.	Ø: 2.64 Σ: 207	53 25.6%	53 25.6%	28 13.53%	61 29.47%	12 5.8%

I recommend that the patient make an independent (private) therapeutic trial of THC-containing cannabis for oral ingestion; re-consultation if there is subsequently a continued desire for medical prescription.	Ø: 3.38 Σ: 206	11 5.34%	30 14.56%	52 25.24%	96 46.6%	17 8.25%
I refer to a recognized cannabis expert so that a treatment trial can be evaluated.	Ø: 2.52 Σ: 208	62 29.81%	63 30.29%	20 9.62%	38 18.27%	25 12.02%
I initiate a therapy trial with cannabis/cannabinoids and submit an application to the BAG (Federal Office of Public Health); documentation according to new regulations.	Ø: 3.33 Σ: 187	19 10.16%	33 17.65%	27 14.44%	84 44.92%	24 12.83%

Case vignette B: Ms. B. 25 y. / "low-risk non-medical cannabis use".

"Ms. B. is a 25-year-old office worker. She is undergoing psychotherapeutic treatment with you due to an adjustment disorder and has no other psychiatric illnesses. She has no children/no desire to have children. Ms. B. reports that she smokes one cannabis joint on 1-2 days per week on average. According to the patient, this consumption has a relaxing and sleep-inducing effect. She only uses tobacco together with cannabis. Ms. B. rarely drinks alcohol, e.g. on social occasions. When Ms. B. consumes cannabis, she does not drink alcohol. No other drugs or substances are consumed. She always keeps her agreed appointments and appears well integrated socially and professionally."

How do you proceed? What are your thoughts regarding Ms. B's cannabis use? Which statements are true for you?

Personal assessment of Ms. B's cannabis use.

	**Ø**	**agree 1**	**rather agree 2**	**rather disagree 3**	**disagree 4**	**do not know / no answer 5**
I currently rate Ms. B's consumption as low risk.	Ø: 1.98 Σ: 206	82 39.81%	74 35.92%	29 14.08%	15 7.28%	6 2.91%

Discontinuing consumption could have a positive effect on Ms. B's overall physical health.	Ø: 2.67 Σ: 206	39 18.93%	62 30.1%	55 26.7%	29 14.08%	21 10.19%
Discontinuing consumption could have a positive impact on Ms. B's overall mental health.	Ø: 2.67 Σ: 206	40 19.42%	65 31.55%	49 23.79%	28 13.59%	24 11.65%

Procedure in relation to case vignette B.

	**Ø**	**agree 1**	**rather agree 2**	**rather disagree 3**	**disagree 4**	**do not know / no answer 5**
I note the consumption and do not elaborate.	Ø: 2.88 Σ: 202	27 13.37%	47 23.27%	55 27.23%	69 34.16%	4 1.98%
I advise against further consumption.	Ø: 2.76 Σ: 205	25 12.2%	63 30.73%	62 30.24%	47 22.93%	8 3.9%
I take this opportunity to make harm reduction recommendations.	Ø: 2.14 Σ: 204	66 32.35%	81 39.71%	27 13.24%	22 10.78%	8 3.92%

In general, what harm reduction recommendations do you feel are useful in relation to cannabis use?

	**Ø**	**agree 1**	**rather agree 2**	**rather disagree 3**	**disagree 4**	**do not know / no answer 5**
Recommending lower-risk forms of consumption in general.	Ø: 2.07 Σ: 203	83 40.89%	73 35.96%	10 4.93%	23 11.33%	14 6.9%

Test without tobacco admixture	Ø: 2.8 Σ: 202	48 23.76%	54 26.73%	26 12.87%	39 19.31%	35 17.33%
Trial with switch to oral intake of cannabis	Ø: 3.07 Σ: 202	35 17.33%	48 23.76%	31 15.35%	43 21.29%	45 22.28%
Referral to a specialized addiction counseling center	Ø: 2.51 Σ: 200	59 29.5%	56 28%	25 12.5%	44 22%	16 8%
