# Supplementary material for: Attitudes of Swiss psychiatrists towards cannabis regulation and medical use in psychiatry: a cross-sectional study
Source: J Cannabis Res. 2023 Dec 6;5:40. doi: 10.1186/s42238-023-00210-y (PMC10699035; doi:10.1186/s42238-023-00210-y)
Supplement: Supplementary file 2 — Additional file 2. Structure of the questionnaire; A table showing the chapters of the survey and the associated questions’ numbers. [file 42238_2023_210_MOESM2_ESM.pdf]

Survey chapter	Questions
Demographic data	1-10
Fictive case report A (Cannabis, CBD and THC for therapy in mental disorders)	11-13
Reservations in general about cannabis for medical purposes in mental disorders	14
Personal attitudes towards cannabis for medical purposes in mental disorders	15
Prevalence estimation and experience with cannabis for medical purposes in mental disorders.	16
Fictive case report B (low-risk non-medical cannabis use)	17-20
Personal attitudes towards legal aspects concerning cannabis for non-medical purposes	21-25
Personal attitudes towards health effects of cannabis and tobacco use	26-31
