# Supplementary material for: Attitudes of Swiss psychiatrists towards cannabis regulation and medical use in psychiatry: a cross-sectional study
Source: J Cannabis Res. 2023 Dec 6;5:40. doi: 10.1186/s42238-023-00210-y (PMC10699035; doi:10.1186/s42238-023-00210-y)
Supplement: Supplementary file 3 — Additional file 3. Demographics of subgroups; A table showing the demographics by canton and the demographics by responder status. [file 42238_2023_210_MOESM3_ESM.pdf]

	n (%) German-speaking cantons*	n (%) French-speaking cantons*	n (%) full responders	n (%) partial responders
**Age**	**n=181**	**n=42**	**n=97**	**n=151**
<30 years	11 (6.1)	2 (4.8)	8 (8.2)	6 (4.0)
30-40 years	43 (23.8)	3 (7.1)	24 (24.7)	26 (17.2)
41-50 years	39 (21.5)	12 (28.6)	24 (24.7)	33 (21.8)
51-60 years	50 (27.6)	13 (30.9)	20 (20.6)	47 (31.1)
>60 years	36 (19.9)	11 (26.2)	20 (20.6)	37 (24.5)
Not specified	2 (1.1)	1 (2.4)	1 (1.0)	2 (1.3)
**Sex**	**n=184**	**n=42**	**n=97**	**n=154**
Women	71 (38.6)	11 (50.0)	37 (38.1)	65 (42.2)
Men	109 (59.2)	20 (47.6)	57 (58.8)	85 (55.2)
Other definition	3 (1.6)	0 (0.0)	3 (3.1)	0 (0.0)
Not specified	1 (0.5)	1 (2.4)	0 (0.0)	4 (2.6)
**Board certified Psychiatrist**	**n=184**	**n=41**	**n=97**	**n=154**
Yes	125 (67.9)	38 (92.7)	71 (73.2)	114 (74.0)
No	59 (32.1)	3 (7.3)	26 (26.8)	40 (26.0)
**Active years in Psychiatry**	**n=184**	**n=41**	**n=97**	**n=151**
<2 years	15 (8.1)	0 (0.0)	8 (8.2)	7 (4.64)
2-5 years	16 (8.7)	3 (7.3)	10 (10.3)	11 (7.3)
5-10 years	29 (15.8)	2 (4.9)	15 (15.5)	17 (11.3)
11-15 years	27 (14.7)	8 (19.5)	18 (18.6)	21 (13.9)
16-20 years	23 (12.5)	10 (24.4)	11 (11.3)	25 (16.6)
>20 years	73 (39.7)	18 (43.9)	35 (36.1)	68 (45.0)
Not specified	1 (0.5)	0 (0.0)	0 (0.0)	2 (1.3)
**Setting**	**n=181**	**n=42**	**n=97**	**n=151**
Own Practice	83 (45.9)	39 (92.9)	48 (49.5)	92 (60.9)
Institution, mainly outpatient	52 (28.7)	1 (2.4)	24 (24.7)	31 (20.5)
Institution, mainly inpatient	44 (24.3)	2 (4.8)	24 (24.7)	26 (17.2)
Not specified	2 (1.1)	0 (0.0)	1 (1.0)	2 (1.3)
