# Supplementary figures and images for: Attitudes of Swiss psychiatrists towards cannabis regulation and medical use in psychiatry: a cross-sectional study
Source: J Cannabis Res. 2023 Dec 6;5:40. doi: 10.1186/s42238-023-00210-y (PMC10699035; doi:10.1186/s42238-023-00210-y)

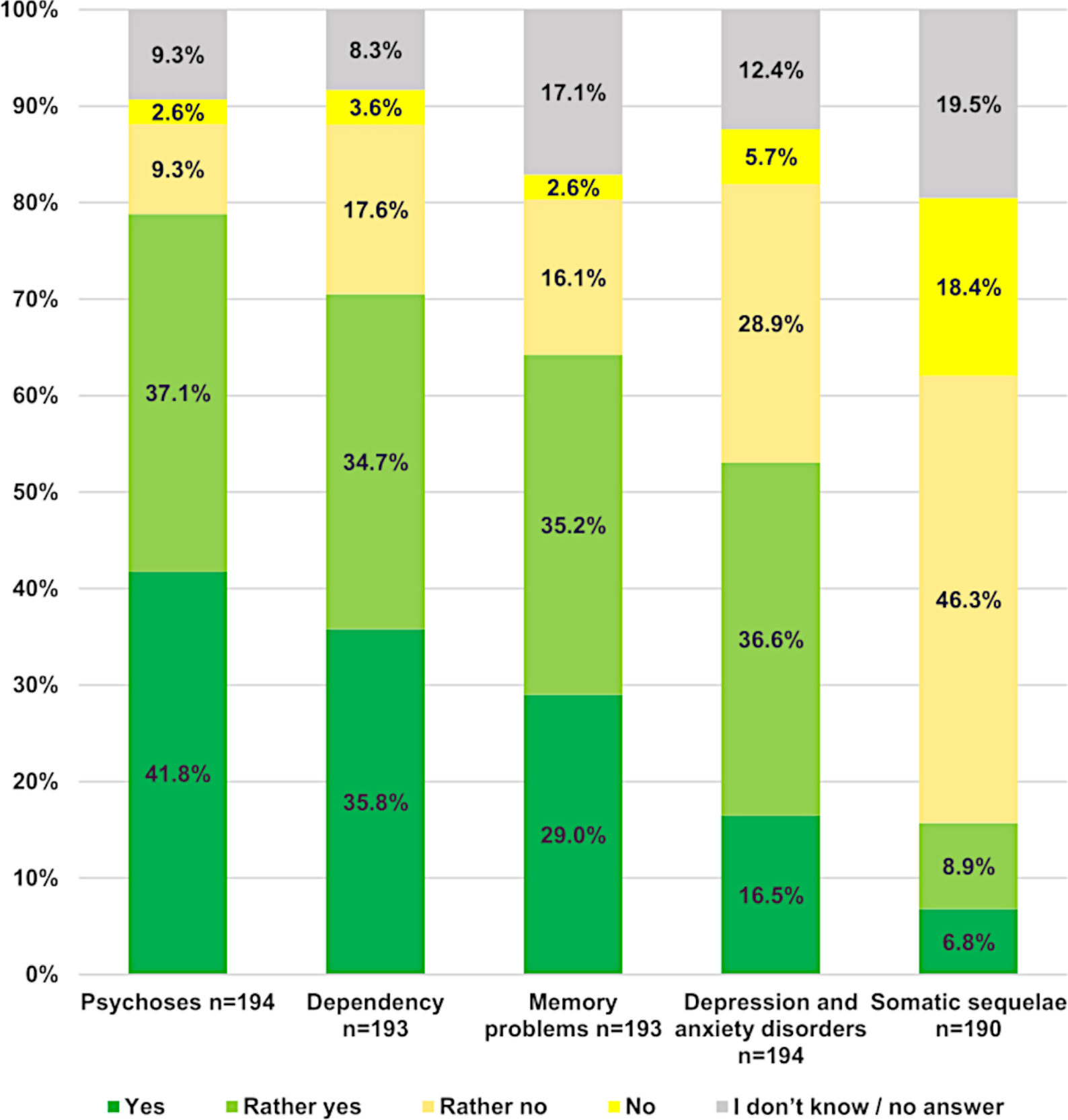

Supplement: Supplementary file 4 — Additional file 4. Swiss psychiatrists’ opinion on emergence of consequential diseases with THC use; A stacked bar chart showing the participants’ opinion of possible consequential diseases associated with consumption of THC. [file 42238_2023_210_MOESM4_ESM.pdf]

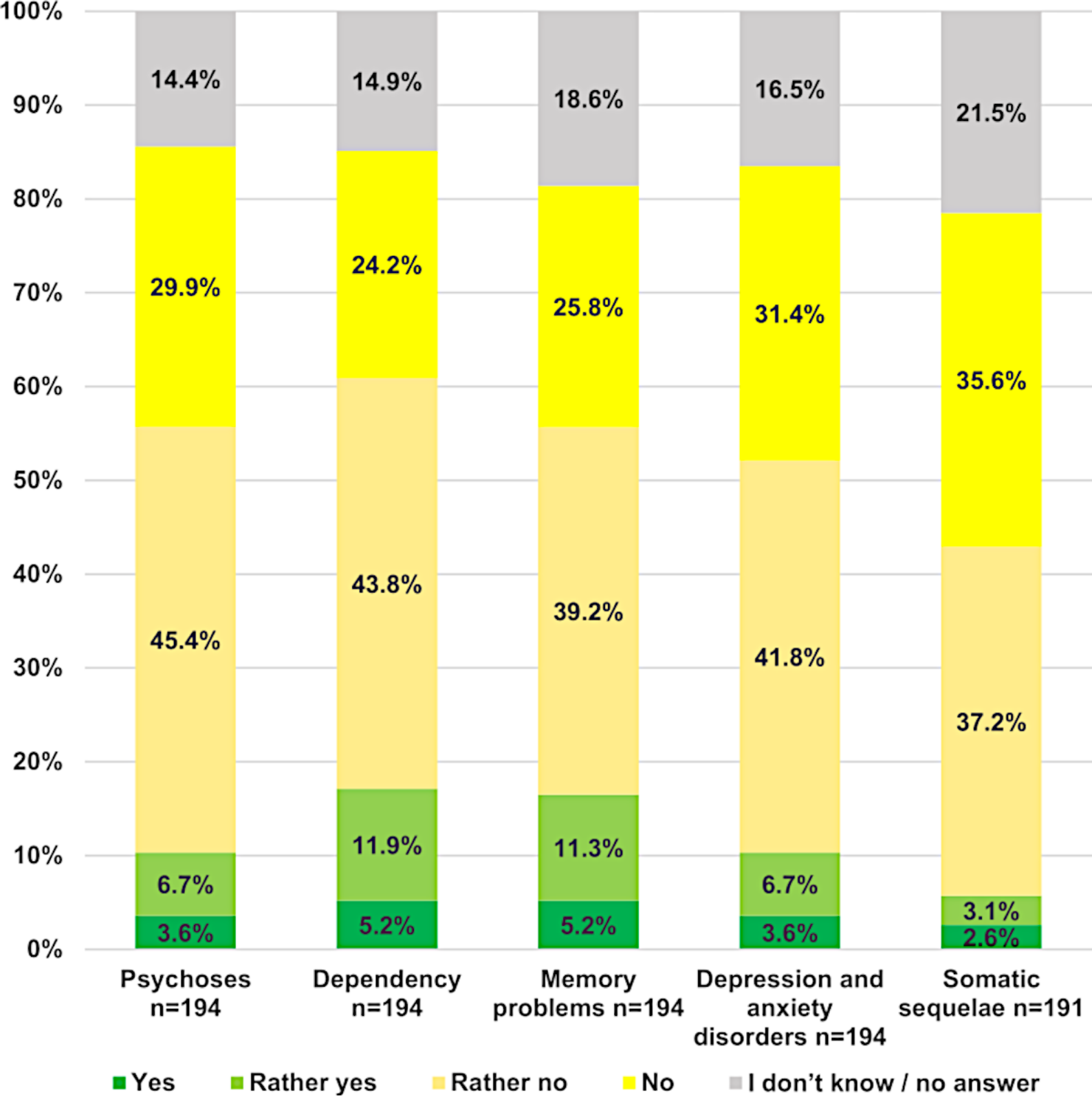

Supplement: Supplementary file 5 — Additional file 5. Swiss psychiatrists’ opinion on emergence of consequential diseases with CBD use; A stacked bar chart showing the participants’ opinion of possible consequential diseases associated with consumption of CBD. [file 42238_2023_210_MOESM5_ESM.pdf]

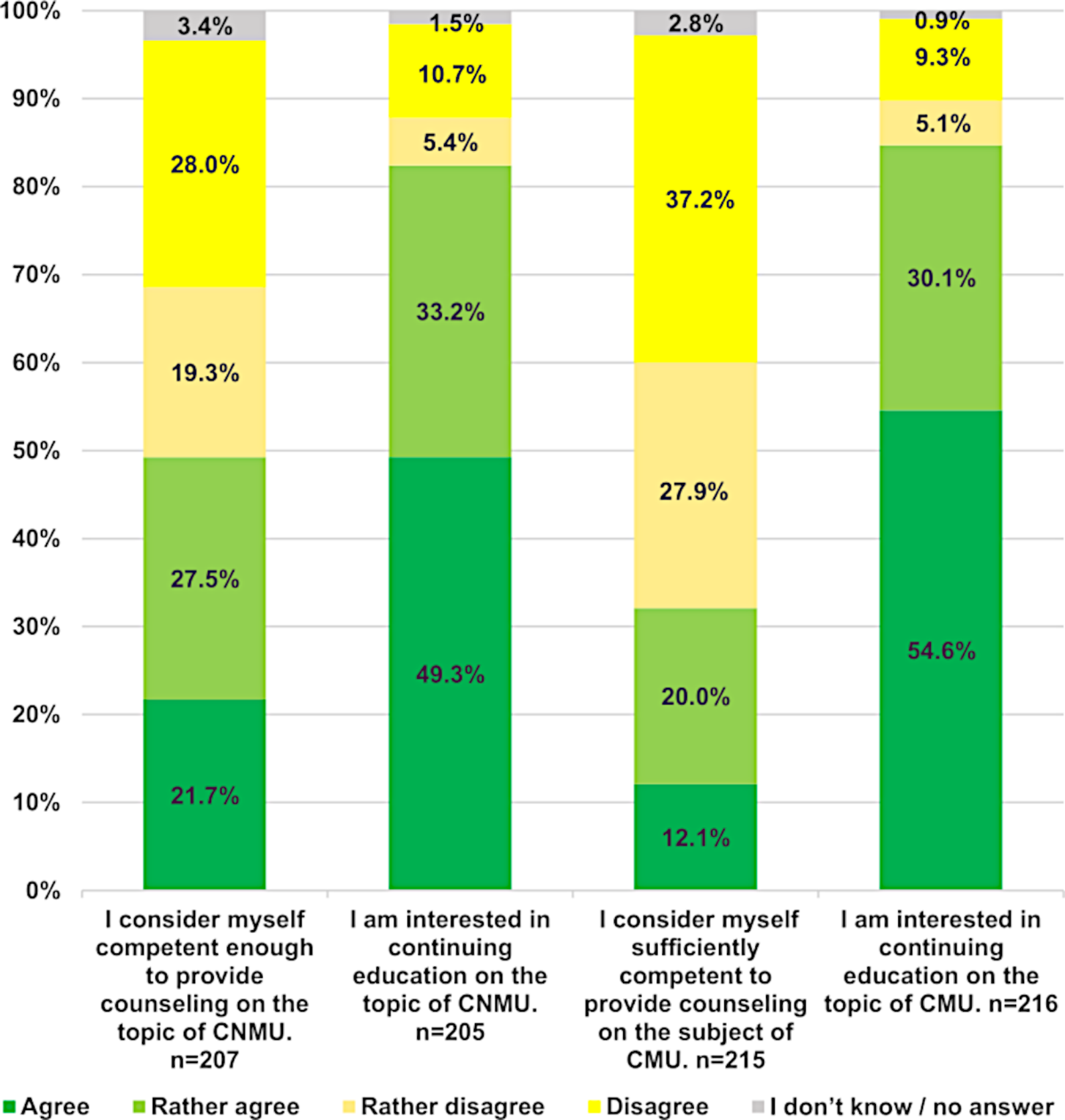

Supplement: Supplementary file 6 — Additional file 6. Competency and interest in continuing education of Swiss psychiatrists concerning CNMU and CMU; A stacked bar chart showing the personally perceived competency and interest of continuing education concerning CNMU and CMU. [file 42238_2023_210_MOESM6_ESM.pdf]

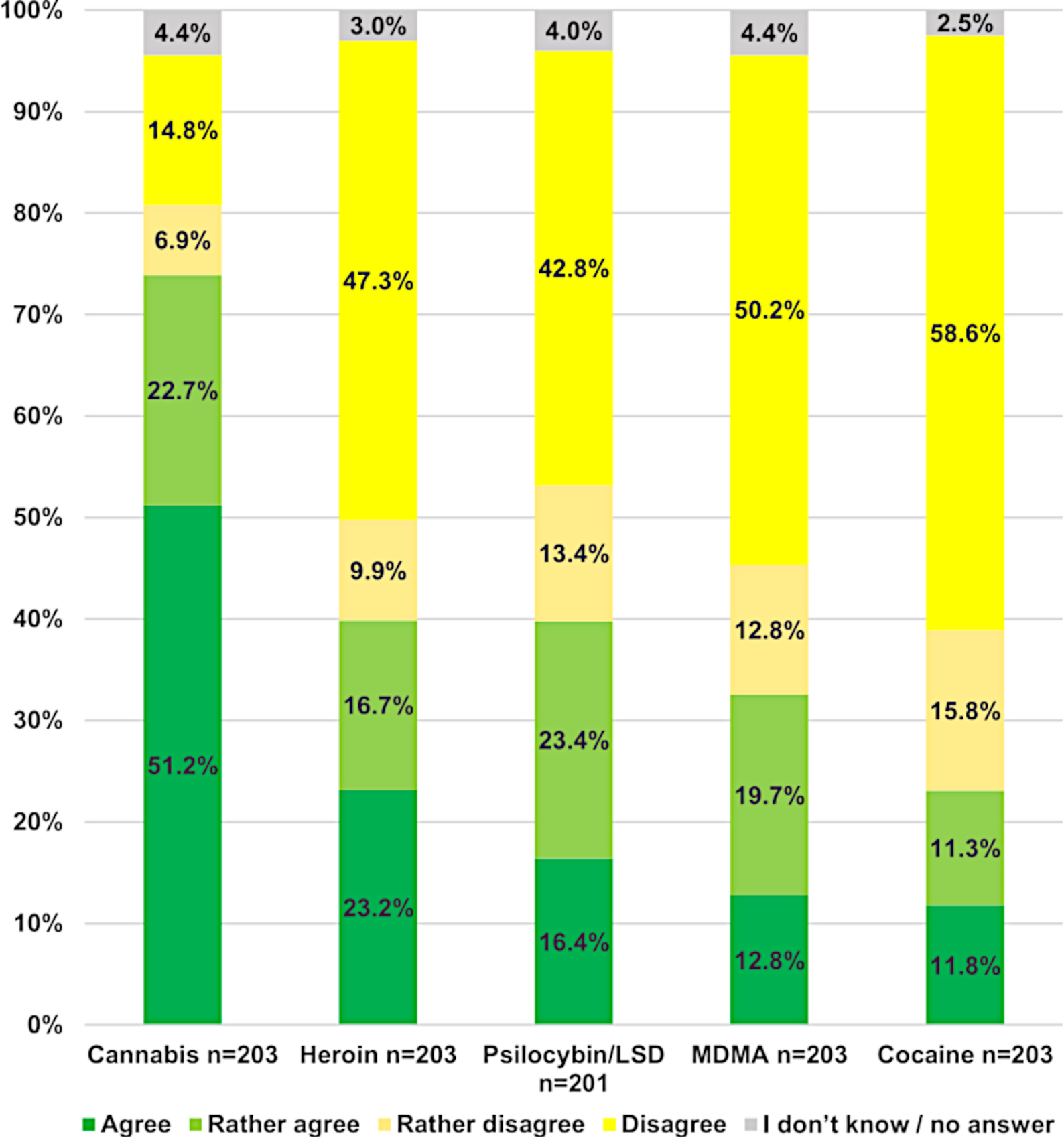

Supplement: Supplementary file 7 — Additional file 7. Support of regulated dispensing of various drugs; A stacked bar chart showing the participants’ support of regulated dispensing of various drugs. [file 42238_2023_210_MOESM7_ESM.pdf]
