# Supplementary material for: Attitudes of Swiss psychiatrists towards cannabis regulation and medical use in psychiatry: a cross-sectional study
Source: J Cannabis Res. 2023 Dec 6;5:40. doi: 10.1186/s42238-023-00210-y (PMC10699035; doi:10.1186/s42238-023-00210-y)
Supplement: Supplementary file 8 — Additional file 8. Bivariate analyses; A table with the quantitative results of the bivariate analyses. [file 42238_2023_210_MOESM8_ESM.pdf]

		Research			Vote	
**Independent variable (type)**	**n**	**z-value**	**p-value**	**n**	**z-value**	**p-value**
Age (<50/>50)	85/95	0.756	0.4650	82/92	0.634	0.5280
Sex (women/men)	71/111	-0.242	0.8333	67/109	1.327	0.1879
Board certification (yes/no)	135/48	1.027	0.3143	133/44	-0.143	0.8898
Active years (<10/>10)	47/136	-0.933	0.3632	47/129	0.977	0.3339
Setting (own practice/institution)	98/83	-0.192	0.8509	100/76	-0.042	0.9656
Canton (German/French)	145/26	-3.032	0.0043*	139/23	-3.104	0.0021*
Responder (complete/partial)	89/96	-0.791	0.4420	85/94	-0.857	0.3999

*Significant on the p<0.05 level
