# Supplementary material for: Attitudes of Swiss psychiatrists towards cannabis regulation and medical use in psychiatry: a cross-sectional study
Source: J Cannabis Res. 2023 Dec 6;5:40. doi: 10.1186/s42238-023-00210-y (PMC10699035; doi:10.1186/s42238-023-00210-y)
Supplement: Supplementary file 9 — Additional file 9. Ordered logistic regression analyses; A table with the quantitative results of the ordered logistic regression analyses. [file 42238_2023_210_MOESM9_ESM.pdf]

Independent variable (type)	Regulation consumption 95% CI	z-value	p-value	Evidence 95% CI	z-value	p-value	Effectiveness 95% CI	z-value	p-value
Age (ordinal)	[-0.29; 0.70]	0.81	0.421	[-0.22; 0.81]	1.12	0.262	[-0.20; 0.79]	1.16	0.244
Sex (categorial)									
Men	[-0.96; 0.26]	-1.12	0.264	[-0.74; 0.53]	-0.32	0.749	[-0.59; 0.69]	0.16	0.876
Other definition	[-2.0; 2.57]	0.23	0.816	[-0.26; 3.80]	1.71	0.087	[-0.82; 3.43]	1.20	0.230
Board certification (binary)	[-1.57; 0.43]	-1.12	0.264	[-1.34; 0.82]	-0.47	0.635	[-0.99; 1.19]	0.17	0.862
Active years (ordinal)	[-0.94; -0.05]	-2.16	0.031*	[-1.07; -0.10]	-2.34	0.019*	[-0.72; 0.19]	-1.13	0.257
Setting (categorial)									
Mainly outpatient	[-0.76; 1.01]	0.28	0.777	[-2.10; -0.23]	-2.43	0.015*	[-2.23; -0.29]	-2.54	0.011*
Mainly inpatient	[-0.99; 1.05]	0.07	0.948	[-3.36; -1.06]	-3.76	<0.001*	[-2.09; 0.00]	-1.95	0.051
Canton (binary)	[-0.28; 1.61]	1.38	0.168	[-1.96; -0.16]	-2.31	0.021*	[-3.05; -1.22]	-4.57	<0.001*
Responder (binary)	[-0.39; 0.84]	0.71	0.479	[-0.62; 0.65]	0.04	0.964	[-0.51; 0.78]	0.40	0.690

*Significant on the p<0.05 level
